# The Role of HLA-Class Ib Molecules in Immune-Related Diseases, Tumors, and Infections 2016

**DOI:** 10.1155/2017/2309574

**Published:** 2017-01-04

**Authors:** Roberta Rizzo, Enrico Fainardi, Nathalie Rouas-Freiss, Fabio Morandi

**Affiliations:** ^1^Department of Experimental and Diagnostic Medicine, Section of Microbiology, University of Ferrara, Ferrara, Italy; ^2^Neuroradiology Unit, Department of Diagnostic Imaging, Azienda Ospedaliero-Universitaria Careggi, Florence, Italy; ^3^CEA, Institut des Maladies Emergentes et des Thérapies Innovantes (iMETI), Service de Recherches en Hémato-Immunologie (SRHI), Hôpital Saint-Louis, Paris, France; ^4^Laboratory of Oncology, G. Gaslini Scientific Institute, Genoa, Italy

HLA-class I family includes highly polymorphic HLA-class Ia molecules (HLA-A, HLA-B, and HLA-C), which play a central role in adaptive immunity, and “nonclassical” HLA-class Ib molecules (HLA-E, HLA-F, HLA-G, and HLA-H), characterized by a limited polymorphism and a few alleles that encode a small number of functional proteins. Both types of HLA-class I molecules can bind peptides generated from cytosolic antigens and present them to specific CD8^+^ T lymphocytes. However, the main function of HLA-class Ib molecules is the modulation of immune responses, both in physiological and in pathological conditions. In contrast, nonclassical major histocompatibility complex (MHC) class I chain related (MIC) molecules show homology with classical human leukocyte antigen (HLA) molecules, but they do not bind beta-2 microglobulin and peptides. Expression of MIC proteins is upregulated on the cell surface in response to stress, and these molecules can interact with the activating natural killer cell receptor NKG2D, which is expressed by many cells of the immune system.

HLA-G, the best characterized HLA-Ib molecule, was firstly detected on fetal cytotrophoblast cells during pregnancy, where it abrogates maternal NK cell activity protecting fetal tissues. However, HLA-G expression and release can be detected in different cells and tissues in pathological conditions, such as tumors, transplantation, bacterial and viral infections, and inflamed tissues. HLA-G can interact with at least four specific receptors (ILT2, ILT4, KIR2DL4, and CD160) that are expressed on T and B lymphocytes, NK cells, neutrophils, and antigen presenting cells, thus inhibiting their functions.

HLA-E is expressed by all nucleated cells and binds peptides derived from the leader sequence of other HLA-class I molecules. In normal conditions, HLA-E interacts with CD94/NKG2A inhibitory receptor on NK cells, thus inhibiting their cytotoxicity against cells expressing HLA-class I molecules. Transformed or virus-infected cells, which usually downregulate HLA-class I expression, display a lower number of HLA-class I-derived peptides. This in turn leads to downregulation of HLA-E expression, thus allowing NK cells lo lyse these cells. However, different transformed cells can upregulate HLA-E expression to avoid NK cell mediated lysis. Finally, it has been demonstrated that HLA-E can also interact with CD94/NKG2C activatory receptor, thus leading to the activation of NK cell functions. This function is important for vascular remodeling during pregnancy.

HLA-F can act as chaperone for the *β*2-microglobulin-free heavy chain of other HLA-class I molecules, and its expression can be detected on the surface of activated lymphocytes. Moreover, it has been demonstrated that this molecule can be present in a peptide-free (open conformer) form and can cooperate with other HLA-class I open conformers during cross-presentation. Finally, HLA-F open conformers can interact with different KIR on NK cells.

No functional HLA-H molecules encoded by HLA-H alleles have been yet characterized.

In this special issue dedicated to nonclassical HLA-class Ib molecules, we have discussed in a review novel findings obtained in the last two years, regarding the role of HLA-G in cancer, infectious diseases, autoimmune/inflammatory diseases, pregnancy, and transplantation.

One interesting paper by Y. Zhang et al. addressed the role of* MICB* in SLE patients. They have demonstrated that a single nucleotide polymorphism (SNP) called rs3828903 is associated with higher susceptibility to systemic lupus erythematosus (SLE), and a higher* MICB *gene expression was detected in SLE patients, thus suggesting a role of this molecule in the progression of the disease.

Three papers have addressed the role of HLA-E in pathological conditions. J. Di Cristofaro et al. have demonstrated that lung transplanted patients that displayed homozygosis for HLA-E^*∗*^01:01 or HLA-E^*∗*^01:03 alleles showed impaired overall survival as compared with patients displaying heterozygosis. Moreover, the presence of HLA-E^*∗*^01:03 allele is correlated with a higher incidence of chronic rejection, as compared with patients with HLA-E^*∗*^01:01 homozygosis. An interesting review by S. A. Joosten et al. discussed the role of pathogen-specific HLA-E restricted T-cell responses during infectious diseases. Finally, F. Morandi et al. have demonstrated that soluble HLA-E concentration was higher in BM plasma samples from neuroblastoma patients with metastatic disease than in those with localized tumors, thus suggesting a role of this molecule in the progression of the disease. The same results have been also obtained for soluble HLA-G.

Three papers have addressed the role of HLA-G in pathological conditions. G. Murdaca et al. have discussed in their review the role of HLA-G during allergic diseases, pointing out the role of this molecule in the suppression of the allergic reaction. R. Rizzo and coworkers have demonstrated that plasma sHLA-G levels were higher in women with primary human congenital cytomegalovirus (HCMV) infection than in nonprimary and uninfected pregnant women. Moreover, sHLA-G levels in amniotic fluid were higher in symptomatic than in asymptomatic fetuses. Collectively, these data suggested a role for HLA-G as a biomarker for HCMV infection. L. L. Olesen and T. V. F. Hviid have characterized the role of HLA-G in left ventricular systolic dysfunction (LVSD). They have demonstrated that soluble HLA-G was higher in patients with ejection fraction (EF) < 50% than in those with EF > 50%. However, sHLA-G was less specific as a biomarker of LVSD than other classic biomarkers. Moreover, a combined 14 bp ins-del/+3142SNP HLA-G haplotype was associated with EF < 40%.

Finally, an interesting review by L. L. Johansen and coworkers discussed the role of classical and nonclassical HLA-class I molecules in the progression of human melanoma.

Collectively, all these papers have added novel important findings on HLA-class Ib molecules in different settings, thus confirming their pivotal role in the control of the immune system both in physiological and in pathological conditions.

*Roberta Rizzo* *Enrico Fainardi* *Nathalie Rouas-Freiss* *Fabio Morandi*

